# Role of recombinant S100A13 expression in regulating mitochondrial fission and fusion in lung epithelial cells

**DOI:** 10.1016/j.bbadva.2026.100195

**Published:** 2026-06-04

**Authors:** Somaya Alqattan, Mona Alonazi, Eid Almutairy, Vineesh V. Raveendran, Abdulaziz Alamri, Futwan Al-mohanna

**Affiliations:** a Department of Biochemistry, College of Science, King Saud University, Riyadh, Saudi Arabia; b Dr. Sulaiman Al Habib Medical Group, As Sahafa Hospital, Riyadh, Saudi Arabia; c Lung Health Centre, King Faisal Specialist Hospital and Research Centre, Riyadh, Saudi Arabia; d College of Medicine, Alfaisal University, Riyadh, Saudi Arabia

**Keywords:** S100A13, p.I80Gfs*13, Calcium transients, Mitochondrial dynamics, Lung epithelial cells, Cristae, ΔΨm

## Abstract

S100A13, a calcium-binding protein containing two EF-hand motifs, contributes to intracellular calcium homeostasis, a key determinant of mitochondrial quality control. We previously showed that S100A13 modulates mitochondrial membrane potential (ΔΨm) in patient-derived skin fibroblasts carrying S100A13 (p.I80Gfs*13*)* and S100A3 (p.R77C) mutations. However, its role in regulating mitochondrial dynamics remains unclear. Here, we investigated whether S100A13 regulates mitochondrial fusion–fission balance in human bronchial epithelial cells (BEAS-2B) using Myc-tagged wild-type or p.I80Gfs*13 S100A13 constructs. The S100A13 p.I80Gfs*13 mutant markedly attenuated bradykinin- and ionophore-induced intracellular calcium transients and reduced ΔΨm compared with wild-type S100A13 (*p* < 0.05). These alterations were associated with severe mitochondrial ultrastructural abnormalities, disrupted cristae organization, and increased mitochondrial fragmentation (*p* < 0.05). Interestingly, both wild-type and p.I80Gfs*13 mutant S100A13 increased expression of the mitochondrial fusion-associated proteins MFN1/2 and OPA1 while reducing expression of the fission mediator MFF. However, despite these apparently pro-fusion molecular changes, the S100A13 p.I80Gfs*13 mutant failed to maintain mitochondrial fusion competency, suggesting a functional uncoupling between fusion protein abundance and mitochondrial fusion competency. Collectively, these findings identify S100A13 as an important regulator of intracellular calcium-dependent mitochondrial dynamics and demonstrate that C-terminal truncation disrupts calcium-dependent regulation of mitochondrial fusion and cristae integrity in lung epithelial cells.

## Highlights

• S100A13 links intracellular Ca²⁺ homeostasis to mitochondrial dynamics in lung epithelial cells.
• C-terminal truncation impairs intracellular Ca²⁺ signaling, reduce mitochondrial ΔΨm, and promotes mitochondrial fragmentation.
• C-terminal truncation disrupts cristae integrity and alters MFN1/2, OPA1, and MFF expression.
• C-terminal truncation functionally uncouples fusion protein expression from effective mitochondrial fusion..
• The C-terminal EF-hand domain is required for mitochondrial structural integrity.

## 1 Introduction

The mitochondrial quality control (MQC) system plays a vital role in preserving mitochondrial function and structural integrity. This system comprises three core processes, namely mitochondrial biogenesis, mitochondrial dynamics, and mitophagy [1]. Among these, mitochondrial dynamics are a central component encompassing the highly regulated and continuous processes of fusion and fission [2], which determine mitochondrial shape, size, length, mass, and intracellular distribution [1,3]. Mitochondrial fusion involves the merging of two or more mitochondria into a single organelle, whereas fission refers to the division of one mitochondrion into smaller units [3]. These opposing processes are tightly regulated by specific sets of proteins [4]. Key regulatory proteins involved in mitochondrial fusion include mitofusins (MFN1/2) and optic atrophy 1 (OPA1) [5]. MFN1 and MFN2 are outer mitochondrial membrane GTPases that drive outer membrane fusion and maintain network integrity [6]. MFN1 provides strong GTPase activity for membrane fusion, whereas MFN2 additionally regulates endoplasmic reticulum–mitochondria contacts and calcium signaling [7]. OPA1, a dynamin-related GTPase localized to the inner mitochondrial membrane, mediates inner-membrane fusion and preserves cristae architecture [8]. OPA1 activity requires intact mitochondrial membrane potential (ΔΨm) [9]. In contrast, mitochondrial fission is mediated by proteins including mitochondrial dynamics proteins of 49 and 51 kDa (MiD49/MiD51), dynamin-related protein 1 (DRP1), fission 1 protein (FIS1), and mitochondrial fission factor (MFF) [10]. Notably, MFF is an outer mitochondrial membrane protein [11] that functions as a receptor to recruit and anchor DRP1 to the mitochondrial membrane surface, where DRP1 assembles into constriction rings to drive membrane scission [12]. A balanced fusion–fission cycle is crucial for mitochondrial function and cellular homeostasis [13]. Structurally, mitochondria consist of two membranes: the outer mitochondrial membrane (OMM) and the inner mitochondrial membrane (IMM) [14]. The IMM forms specialized invaginations known as cristae [15]. Cristae remodeling is tightly regulated by intracellular calcium flux [Ca²⁺] [16]. Moreover, mitochondria possess intermembrane space (IMS) between the OMM and IMM [14], along with the mitochondrial matrix that contains enzymes involved in the tricarboxylic acid (TCA) cycle, mitochondrial DNA (mtDNA), and ribosomes [17].

We previously identified co-inherited mutations in S100A3 (c.229C>T; p.R77C) and S100A13 (c.238_241delATTG; p.I80Gfs*13) in patients with atypical pulmonary fibrosis [18]. The S100A13 mutation introduces a frameshift at codon 80, which likely generates a truncated protein lacking the C-terminal EF-hand domain required for calcium binding. These digenic variants were associated with reduced expression of both proteins and impaired receptor-dependent calcium changes in patient-derived cells. At the mitochondrial level, patient fibroblasts exhibited increased mitochondrial mass, abnormal morphology, and disrupted cristae structure [18]. In a subsequent study, we confirmed that restoration of wild-type S100A13 normalized ΔΨm and reversed mitochondrial hyperpolarization in patient-derived cells. Notably, this effect was accompanied by a reduction in mitochondrial mass and partial restoration of mitochondrial structural organization, thereby indicating improved mitochondrial functional integrity. In contrast, S100A3 alone had no measurable impact on mitochondrial polarization or mass. These findings support a dominant and specific role for S100A13 in coupling calcium-dependent signaling to mitochondrial regulation and quality control mechanisms [19]. However, the role of S100A13 in mitochondrial regulation within lung epithelial cells, which are the primary functional cells of lung tissue, remains poorly defined. Thus, the present study investigated whether S100A13 regulates mitochondrial dynamics in lung epithelial cells. We hypothesized that S100A13 directly or indirectly modulates mitochondrial fusion–fission balance through a calcium-dependent mechanism mediated by its C-terminal domain, and that disruption of the C-terminal EF-hand domain weakens this regulatory axis. Unlike our prior fibroblast-based studies, which demonstrated associations between S100A13 mutations, altered calcium response, and mitochondrial dysfunction, this work focuses on elucidating the mechanistic effects of recombinant wild-type and p.I80Gfs*13 mutant S100A13 on mitochondrial ultrastructure and dynamics in lung epithelial cells.

## 2 Materials and methods

### 2.1 Cell culture and preparation

The human bronchial epithelial cell line BEAS-2B (ATCC CRL-9609) was obtained from the American Type Culture Collection (ATCC, Manassas, VA, USA). Cells were cultured at 37 °C in a humidified incubator with 5% CO₂ in DMEM medium (Cat. No D5796, Sigma-Aldrich) supplemented with 10% bovine calf serum (Cat. No N4762, Sigma-Aldrich) and 1% antibiotic-antimycotic solution (Cat. No A5995, Sigma-Aldrich). Cell viability was determined using the Trypan Blue Solution, 0.4% (Cat. No 15,250,061, Gibco). Additionally, cell maintenance and passaging were carried out following standard cell culture protocols to ensure optimal growth conditions. A mycoplasma test was performed to confirm the absence of mycoplasma contamination.

### 2.2 S100A13 expression

Polymerase Chain Reaction (PCR) was applied to amplify the coding sequences of both wild-type and p.I80Gfs*13 mutant S100A13 using the forward primer 5′-ATGGCAGCAGAACCACTGAC-3′ and the reverse primer 5′-CTTCTTCCTGATCTTCAG-3′ as previously described [19]. The amplified products were cloned into multiple cloning sites of the gWIZ Blank Mammalian Expression Vector (Cat. #P000200, Genlantis, Gene Therapy Systems, Inc.), which was downstream of the CMV promoter. Subsequently, the inserted S100A13 sequences were engineered to include an N-terminal Myc tag (EQKLISEEDL), resulting in fusion proteins consisting of 123 amino acids. Wild-type and mutant plasmids were verified by DNA sequencing [19].

### 2.3 Transfection of cultured cells

BEAS-2B cells (ATCC CRL-9609) were seeded onto 6-well plates or sterile coverslips and incubated overnight to allow adherence. Transfections were carried out using the Lipofectamine 3000 Kit (Cat. #L3000–015, Lot #2041,107; Invitrogen, Thermo Fisher Scientific, USA) according to the manufacturer’s instructions. Briefly, 3 µg of plasmid DNA was used per well for 4–5 × 10⁵ cells, including 500 ng of either red fluorescent protein (RFP) or green fluorescent protein (GFP) expression plasmid as a reporter construct. Transfection efficiency was monitored by co-transfection with GFP or RFP plasmids, and only experiments achieving 60–70% efficiency at 24 h post-transfection were considered for further analyses. For the experimental groups, cells were transfected with Myc-tagged S100A13 constructs encoding either wild-type or p.I80Gfs*13 mutant S100A13, together with RFP- or GFP-expressing vectors. The parallel vector-transfected control (vector control) group was transfected with RFP or GFP plasmid alone. Successful expression of wild-type and mutant S100A13 constructs was confirmed by immunoblotting analyses (Supplementary Figure S1).

### 2.4 Assessment of intracellular calcium transients and mitochondrial structural integrity

Intracellular calcium transients were measured as previously described [18,19] in BEAS-2B cells (ATCC CRL-9609) transfected with wild-type or p.I80Gfs*13 mutant S100A13. Receptor-mediated changes in intracellular fluorescence in response to bradykinin (BK) (Sigma-Aldrich; 50 µM) and calcium ionophore (Iono) (Sigma-Aldrich; 2 µM) were monitored using a Zeiss LSM 510 META laser scanning confocal microscope (Carl Zeiss MicroImaging, GmbH, Germany).

Further, mitochondrial structural integrity was evaluated by quantifying mitochondrial length and performing ultrastructural assessment. For mitochondrial length measurements, cells were fixed in 3.75% formaldehyde for 15 min and subsequently permeabilized with 0.5% Triton X-100 for 5 min. Mitochondria were stained with MitoTracker Red CMXRos (Cat. No M7512, Invitrogen) at a final concentration of 0.5 µM for 10 min at 20–25 °C, followed by three washes with phosphate-buffered saline (PBS, pH 7.2). MitoTracker™ Red CMXRos is a chloromethyl-X-rosamine–based red fluorescent dye. Fluorescence images were captured using a Carl Zeiss fluorescence microscope, and mitochondrial length was measured using Fiji (ImageJ) by tracing individual mitochondrial filaments from the acquired images. For transmission electron microscope (TEM) analysis, BEAS-2B cells (ATCC CRL-9609) co-transfected with wild-type or p.I80Gfs*13 mutant were fixed in 1.6% glutaraldehyde and post-fixed in 1% osmium tetroxide (OsO₄). Samples were dehydrated through a graded acetone series, embedded in resin using BEEM capsules, and polymerized overnight at 70 °C. Finally, ultrathin sections were examined using a JEM-1400 microscope.

### 2.5 Estimation of mitochondrial mass

Trypsinized BEAS-2B cells (ATCC CRL-9609) (10⁶ cells/mL), co-transfected with wild-type, p.I80Gfs*13 mutant, or vector control, were incubated with MitoTracker Green FM (Cat. # M-7514, Lot #3281–2; Molecular Probes) at a final concentration of 1 µM for 15 min. Following staining, cells were washed with PBS and re-suspended in complete DMEM culture medium. Mitochondrial fluorescence intensity was analyzed using a FACSCalibur flow cytometer (BD Biosciences). Flow cytometry data were analyzed using a sequential gating strategy to exclude debris and doublets prior to fluorescence quantification.

### 2.6 Quantification of expression of S100A13, MFN1/2, OPA1, MFF, and GAPDH

Western blotting was performed as previously described [18]. Briefly, BEAS-2B cells (ATCC CRL-9609), both co-transfected and non-transfected, were lysed and probed with primary antibodies against S100A13 (Cat. No ab109252, Abcam), MFN1/2 (Cat. No ab57602, Abcam), OPA1 (Cat. No ab119685, Abcam), MFF (Cat. No ab129075, Abcam), and GAPDH (Cat. No ab8245, Abcam). Thereafter, blots were incubated with HRP-conjugated secondary antibodies, Goat anti-rabbit IgG-HRP (Cat. No A0545, Sigma-Aldrich), or Goat anti-mouse IgG-HRP (Cat. No A4416, Sigma-Aldrich) according to the manufacturers’ instructions. Primary antibodies against MFN1/2, OPA1, and MFF were used at a dilution of 1:700, S100A13 at 1:500, and GAPDH at 1:800. HRP-conjugated secondary antibodies were used at a dilution of 1:10,000. Bands were visualized using enhanced chemiluminescence (ECL) detection. Band intensities were quantified by densitometric analysis using ImageJ software (NIH, Bethesda, MD, USA) and normalized to GAPDH.

For immunofluorescence, cells were fixed with 3.75% formaldehyde, permeabilized with 0.5% Triton X-100, and blocked with 5% BSA. Cells were incubated with primary anti-S100A13 antibodies (Cat. No ab109252, Abcam) for 2 h at 20–25 °C, followed by incubation with fluorescein-conjugated anti-rabbit IgG secondary antibodies (Cat. No A-11,034, Thermo Fisher Scientific) for 1 h at 20–25 °C according to the manufacturer’s instructions. Primary anti-S100A13 antibody was used at a dilution of 1:100, and the corresponding secondary antibody was used at 1:400 dilution.

### 2.7 Assessment of mitochondrial membrane potential

Following culture and transfection, BEAS-2B cells (ATCC CRL-9609) were trypsinized, washed with PBS, and incubated with MitoTracker Red CMXRos (Cat. No M7512, Invitrogen) at a final concentration of 50 nM for 20 min at 20–25 °C in the dark. Subsequently, cells were washed, resuspended in PBS, and immediately analyzed by flow cytometry. Thereafter, flow cytometry data were analyzed using a sequential gating strategy based on FSC/SSC parameters and singlet discrimination to exclude debris and doublets prior to fluorescence quantification.

### 2.8 Statistical analysis

Statistical analyses were performed using GraphPad Prism (version 10; GraphPad Software, La Jolla, CA, USA). Quantitative data were analyzed using one-way analysis of variance (ANOVA) followed by Tukey’s multiple-comparison test. For comparisons between two independent groups, unpaired two-tailed Student’s *t*-tests were applied where appropriate. Qualitative (categorical) data were analyzed using the Chi-square test or Fisher’s exact test, as appropriate. Notably, a p-value ≤ 0.05 was considered statistically significant. All experiments were performed in at least three independent biological replicates.

## 3 Results

### 3.1 The S100A13 p.I80Gfs*13 mutation impairs intracellular calcium mobilization

**Fig. 1:**
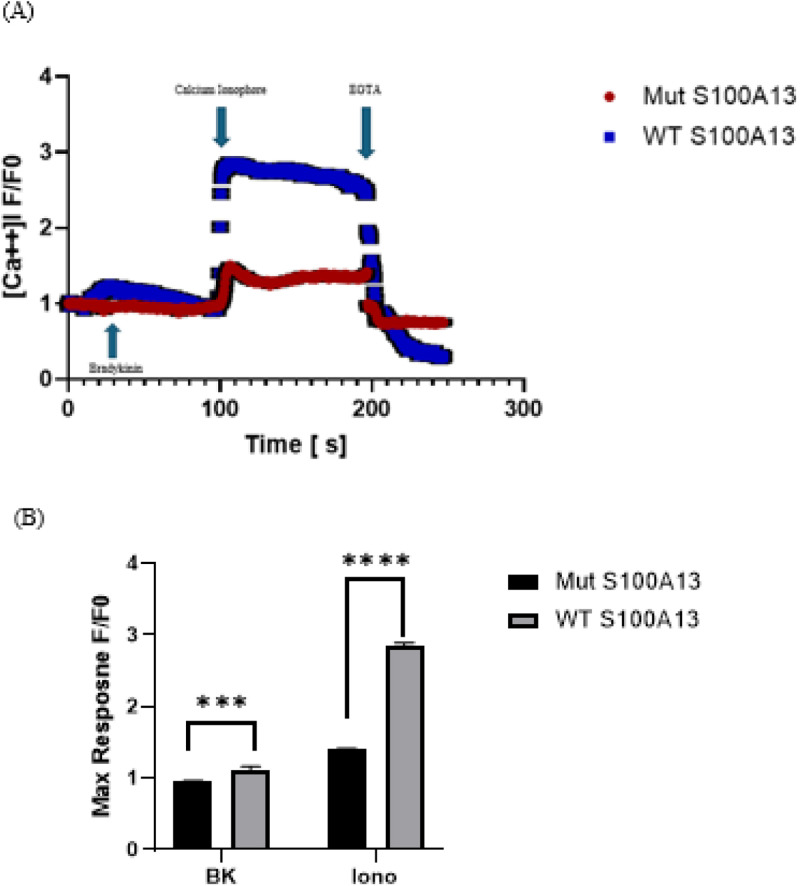
Mutant S100A13 p.I80Gfs*13 Impairs Intracellular Calcium Transients in BEAS-2B Cells. (A) Representative intracellular [Ca²⁺] traces in BEAS-2B cells expressing mutant S100A13 (Mut S100A13; red) or wild-type S100A13 (WT S100A13; blue) following sequential stimulation with bradykinin (50 µM), calcium ionophore (2 µM), and EGTA (1 µM) in calcium-containing Krebs buffer. Arrows indicate the time points of stimulation. (B) Quantification of peak intracellular [Ca²⁺] responses following bradykinin and ionophore stimulation. Data are presented as normalized fluorescence intensity (F/F₀), where F₀ represents baseline fluorescence intensity. WT S100A13–expressing cells exhibited significantly higher intracellular [Ca²⁺] responses than Mut S100A13–expressing cells (*p < 0.05). Data are presented as mean ± SEM from at least three independent experiments (n = 24 cells). Statistical analysis was performed using an unpaired two-tailed Student’s t-test.

Expression of the S100A13 p.I80Gfs*13 mutant significantly impaired intracellular [Ca²⁺] responses compared with the wild-type group (Fig. 1). S100A13 p.I80Gfs*13–expressing cells exhibited significantly reduced bradykinin-induced and ionophore-stimulated intracellular [Ca²⁺] responses compared with wild-type S100A13–expressing cells (*p* < 0.05). Intracellular calcium dynamics are tightly coupled to ΔΨm [20]. Therefore, impaired intracellular calcium mobilization likely contributes to mitochondrial depolarization in S100A13 p.I80Gfs*13–expressing cells.

### 3.2 The S100A13 p.I80Gfs*13 mutation impairs mitochondrial membrane potential (ΔΨm)

**Fig. 2:**
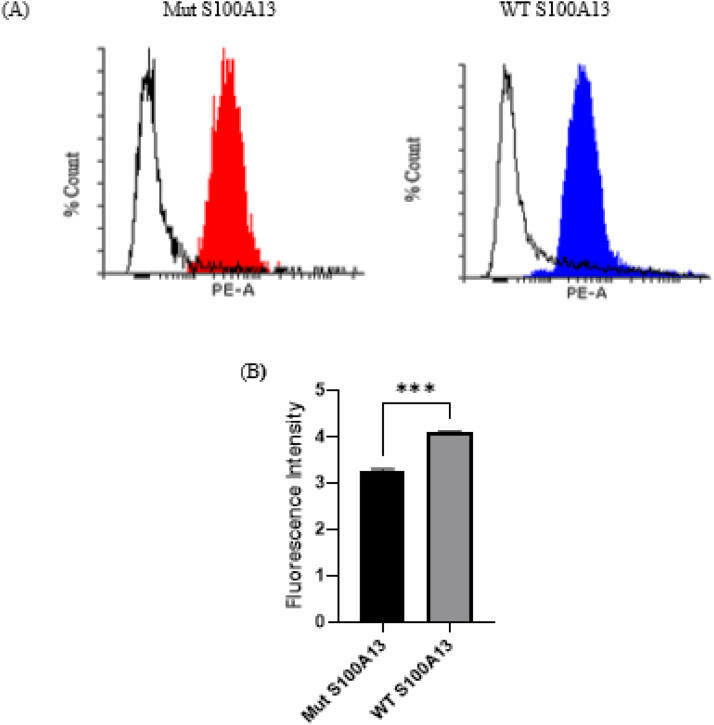
Mutant S100A13 p.I80Gfs*13 Reduces Mitochondrial Membrane Potential in BEAS-2B Cells. (A) Representative overlay histograms of MitoTracker™ Red CMXRos fluorescence intensity in mutant S100A13–expressing cells (Mut S100A13; red) and wild-type S100A13–expressing cells (WT S100A13; blue) compared with corresponding unstained controls (black). (B) Quantitative analysis of mitochondrial fluorescence intensity expressed as geometric mean fluorescence intensity (gMFI). WT S100A13–expressing cells exhibited significantly higher mitochondrial membrane potential (ΔΨm) compared with Mut S100A13–expressing cells (***p < 0.001). Data are presented as mean ± SEM from three independent experiments. Statistical significance was determined using an unpaired two-tailed Student’s t-test.

Expression of the S100A13 p.I80Gfs*13 mutant was associated with a significant reduction in ΔΨm compared with the wild-type group (Fig. 2). Quantitative analysis confirmed that mutant cells exhibited significantly lower fluorescence intensity than wild-type cells (*p* < 0.05).

Stability of ΔΨm is critical for proper OPA1-mediated fusion and maintenance of cristae structural integrity [21]. Consistent with this, S100A13 p.I80Gfs*13–expressing cells exhibited marked mitochondrial ultrastructural abnormalities associated with ΔΨm destabilization.

### 3.3 The S100A13 p.I80Gfs*13 mutation disrupts mitochondrial ultrastructural integrity

**Fig. 3:**
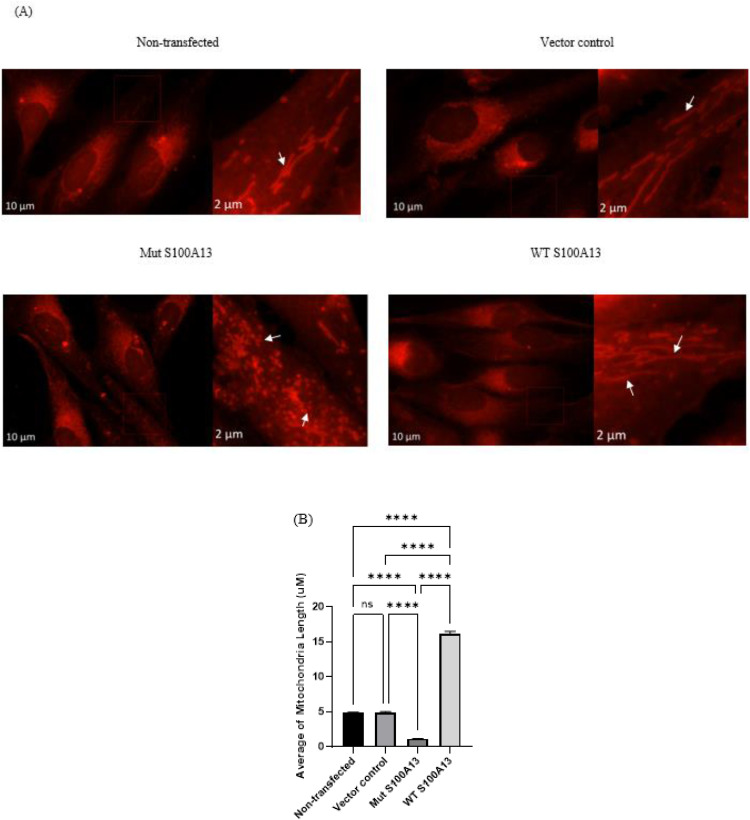
Mutant S100A13 p.I80Gfs*13 Disrupts Mitochondrial Ultrastructural Integrity in BEAS-2B Cells. (A) Representative fluorescence images of mitochondrial morphology in non-transfected, vector control, mutant S100A13–expressing (Mut S100A13), and wild-type S100A13–expressing (WT S100A13) BEAS-2B cells stained with MitoTracker™ Red CMXRos. WT S100A13–expressing cells exhibited elongated and interconnected mitochondrial networks, whereas Mut S100A13–expressing cells displayed fragmented and punctate mitochondrial morphology. Scale bars: 10 µm (low magnification) and 2 µm (high magnification). (B) Quantification of mitochondrial length measured using Fiji (ImageJ). Mut S100A13–expressing cells exhibited significantly reduced mitochondrial length, indicative of mitochondrial fragmentation, whereas WT S100A13–expressing cells showed significantly increased mitochondrial elongation compared with mutant and control cells (*p < 0.05; n = 300 mitochondria). Images are representative of at least three independent experiments.

**Fig. 4:**
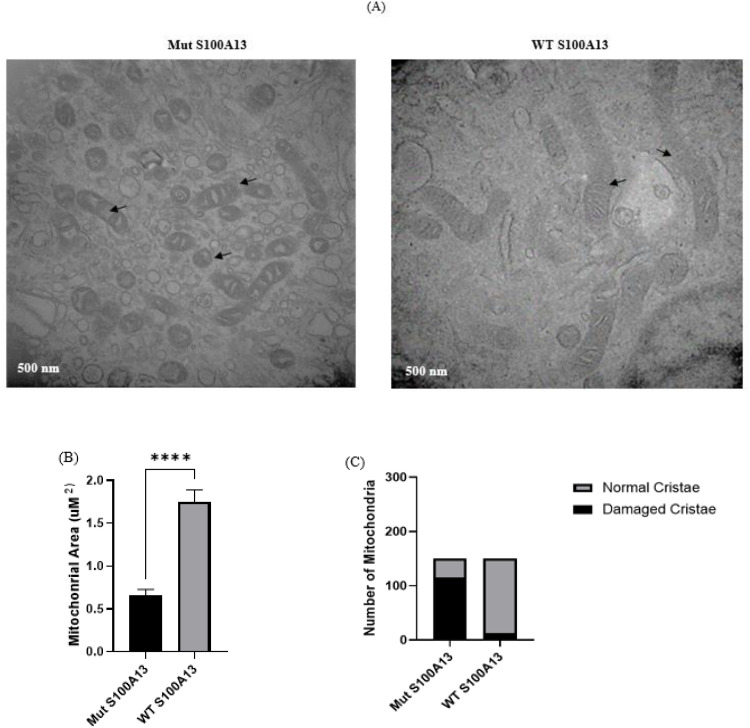
Mutant S100A13 p.I80Gfs*13 Disrupts Mitochondrial Cristae Integrity in BEAS-2B Cells. (A) Representative transmission electron microscopy (TEM) images of BEAS-2B cells expressing mutant S100A13 (Mut S100A13) or wild-type S100A13 (WT S100A13). Mut S100A13–expressing cells exhibited swollen and structurally disorganized mitochondria with disrupted and poorly defined cristae architecture (arrows). In contrast, WT S100A13–expressing cells displayed elongated mitochondria with preserved inner membrane organization and well-defined cristae structure (arrows). Scale bar: 500 nm. Images are representative of at least three independent experiments. (B) Quantification of mitochondrial cross-sectional area derived from TEM images showed that WT S100A13–expressing cells exhibited significantly larger mitochondrial area compared with Mut S100A13–expressing cells (*p < 0.05). (C) Quantification of cristae integrity revealed that Mut S100A13–expressing cells exhibited a significantly higher proportion of damaged cristae compared with WT S100A13–expressing cells, whereas WT cells exhibited a greater proportion of intact cristae (*p < 0.05). Data are presented as mean ± SEM from at least three independent experiments. Statistical analyses were performed using an unpaired two-tailed Student’s t-test for mitochondrial area (B) and Chi-square or Fisher’s exact test for cristae integrity (C).

Expression of the S100A13 p.I80Gfs*13 mutant induced marked mitochondrial ultrastructural abnormalities characterized by mitochondrial swelling, fragmentation, abnormal IMM morphology, and disrupted cristae organization. Mitochondrial length was significantly reduced in S100A13 p.I80Gfs*13–expressing cells compared with wild-type and control cells (*p* < 0.05) (Fig. 3). Ultrastructural analysis by TEM revealed severe cristae disruption in S100A13 p.I80Gfs*13–expressing cells. Interestingly, overexpression of wild-type S100A13 in lung epithelial cells maintained mitochondrial structural integrity, as evidenced by elongated mitochondrial networks and preserved cristae architecture. Mitochondrial cross-sectional area was significantly greater in wild-type cells than mutant cells (***p < 0.0001). Moreover, S100A13 p.I80Gfs*13–expressing cells exhibited a marked reduction in normal cristae (*p* < 0.05) and a substantial increase in cristae damage (Fig. 4; Table 1). These mitochondrial structural abnormalities were accompanied by increased mitochondrial mass in S100A13 p.I80Gfs*13–expressing cells.

**Table 1:** Quantification of mitochondrial cristae integrity in BEAS-2B cells expressing mutant or wild-type S100A13.

Group	Percentage (%) of Damaged Cristae	Percentage (%) of Normal Cristae
Mutant S100A13–expressing cells (Mut S100A13)	76.67%	23.33%
Wild-type S100A13–expressing cells (WT S100A13)	8.67%	91.33%

Cristae integrity was assessed by transmission electron microscopy (TEM): damaged cristae were defined as mitochondria exhibiting disrupted or poorly organized cristae structures. Mutant S100A13–expressing cells exhibited a significantly higher proportion of damaged cristae compared with wild-type cells (*p* < 0.0001).

Statistical analysis was performed using the Chi-square test with Fisher’s exact correction. Data are representative of at least three independent experiments (n = 150 mitochondria).

### 3.4 The S100A13 p.I80Gfs*13 mutation increases mitochondrial mass

**Fig. 5:**
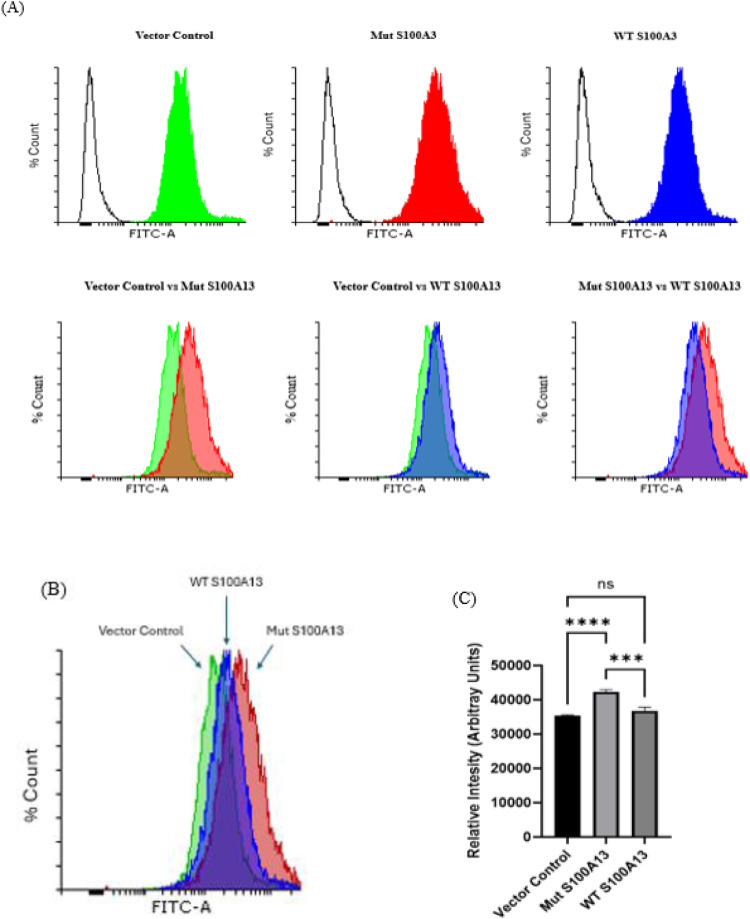
Mutant S100A13 p.I80Gfs*13 Increases Mitochondrial Mass in BEAS-2B Cells. (A) Representative flow cytometry histograms of BEAS-2B cells before and after staining with MitoTracker™ Green FM (1 µM). Gray/black histograms represent unstained controls. A rightward shift in fluorescence intensity in stained samples confirms mitochondrial labeling. (B) Overlay histograms compare fluorescence distributions among vector control, mutant S100A13–expressing (Mut S100A13), and wild-type S100A13–expressing (WT S100A13) cells. Rightward shifts indicate relative increases in mitochondrial content. (C) Quantitative analysis of mitochondrial mass based on FITC-channel fluorescence intensity (arbitrary units). Mut S100A13–expressing cells exhibited significantly higher mitochondrial mass compared with both vector control and WT S100A13–expressing cells, whereas WT S100A13–expressing cells showed no significant difference compared with vector control. Data are presented as mean ± SEM from at least three independent experiments. Statistical analysis was performed using one-way ANOVA followed by Tukey’s multiple comparisons test. Significance levels are indicated as ***p < 0.001 and ****p < 0.0001. The inset shows geometric mean fluorescence intensity (gMFI) values. Approximately 10⁶ cells were analyzed per sample. FITC, fluorescein isothiocyanate.

A significant increase in mitochondrial mass was observed in S100A13 p.I80Gfs*13–expressing cells compared with both wild-type S100A13–expressing cells and vector controls (*p* < 0.05) (Fig. 5). Meanwhile, wild-type S100A13–expressing cells exhibited no significant difference compared with vector controls (*p* > 0.05).

Impaired calcium homeostasis, reduced ΔΨm, disrupted cristae organization, and increased mitochondrial fragmentation collectively indicate impaired mitochondrial fusion competency in S100A13 p.I80Gfs*13–expressing cells. Increased mitochondrial mass in mutant cells was further associated with altered expression of key mitochondrial dynamics regulators, including MFN1/2, OPA1, and MFF.

### 3.5 The S100A13 p.I80Gfs*13 mutation functionally uncouples fusion protein expression from effective mitochondrial fusion

**Fig. 6:**
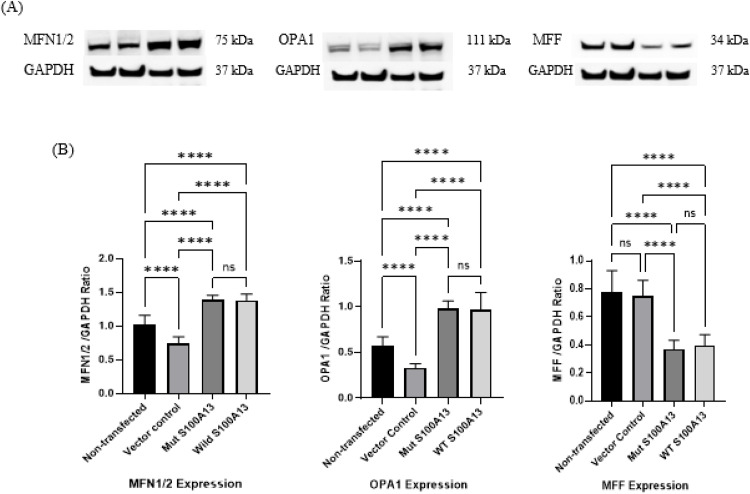
Mutant S100A13 p.I80Gfs*13 Alters Expression of MFN1/2, OPA1, and MFF in BEAS-2B Cells. (A) Representative western blots of mitochondrial dynamics–related proteins in BEAS-2B lung epithelial cells that were non-transfected or transfected with vector control, mutant S100A13 (Mut S100A13), or wild-type S100A13 (WT S100A13). Representative immunoblots showing expression of the mitochondrial fusion proteins Mitofusin 1/2 (MFN1/2; ∼75 kDa) and Optic Atrophy 1 (OPA1; ∼111 kDa), and the mitochondrial fission protein Mitochondrial Fission Factor (MFF; ∼34 kDa). GAPDH (∼37 kDa) was used as a loading control. (B) Densitometric quantification of MFN1/2, OPA1, and MFF protein expression normalized to GAPDH and expressed as relative values. Both Mut S100A13– and WT S100A13–expressing cells exhibited significantly increased MFN1/2 and OPA1 expression, whereas MFF expression was significantly reduced compared with vector control and non-transfected cells (*p < 0.05). Data are presented as mean ± SD from at least three independent experiments. Statistical analysis was performed using one-way ANOVA followed by Tukey’s multiple comparisons test.

Interestingly, both wild-type and p.I80Gfs*13 mutant S100A13 significantly increased the expression of mitochondrial fusion–associated proteins, MFN1/2 (∼75 kDa) and OPA1 (∼111 kDa), while concomitantly reducing expression of the mitochondrial fission mediator MFF (∼34 kDa) compared with controls (*p < 0.05 vs. control) (Fig. 6). However, these molecular changes were not consistent with the mitochondrial morphological phenotype observed in S100A13 p.I80Gfs*13–expressing cells. This discrepancy indicates a functional uncoupling between the abundance of fusion machinery and the execution of mitochondrial fusion competency, suggesting that maintenance of intracellular [Ca²⁺] homeostasis is essential for mitochondrial fusion competency.

In summary, this study demonstrates that the C-terminal EF-hand domain of S100A13 plays an essential role in regulation of mitochondrial dynamics through maintenance of intracellular calcium homeostasis. Loss of the C-terminal EF-hand domain impaired intracellular calcium mobilization, which subsequently led to reduction of ΔΨm. Reduced ΔΨm may impair OPA1 functional activity, which is critical for preservation of cristae structural integrity and maintenance of mitochondrial fusion competency. In addition, impairment of intracellular calcium homeostasis may adversely affect MFN1/2- and MFF-associated mitochondrial fusion–fission regulation. Consequently, these alterations contribute to mitochondrial fragmentation and disruption of mitochondrial structural integrity.

## 4 Discussion

The prevalence of pulmonary diseases, including pulmonary fibrosis (PF), chronic obstructive pulmonary disease (COPD), acute lung injury (ALI), and pulmonary hypertension (pH), continues to rise [22]. Previous studies have demonstrated that mitochondrial dysfunction is a principal driver of pulmonary disease pathogenesis [18,19,22,23], with a key contributing factor being disruption of the MQC system, particularly the imbalance between mitochondrial fusion and fission processes [18,19,23].

In the present study, we identified S100A13—acting through modulation of intracellular [Ca²⁺] homeostasis—as a previously unrecognized regulator of mitochondrial dynamics in lung epithelial cells. Our findings demonstrate that wild-type S100A13 stabilizes ΔΨm, increases expression of MFN1/2 and OPA1 while reducing MFF expression, maintains mitochondrial architecture, preserves cristae integrity, and promotes a fusion-competent mitochondrial network. In contrast, loss of the C-terminal EF-hand domain of S100A13 disrupts intracellular [Ca²⁺] transients, leading to reduced ΔΨm, abnormal mitochondrial morphology, cristae disorganization, and a shift toward a fission-dominant phenotype. These results establish a mechanistic link between S100A13-mediated cytosolic calcium regulation and mitochondrial dynamics.

Intracellular [Ca²⁺] homeostasis is a fundamental determinant of mitochondrial function [24]. Cristae remodeling is tightly regulated by intracellular calcium flux [16,18,19]. In this context, our data confirm that S100A13 modulates intracellular [Ca²⁺] homeostasis. In wild-type S100A13–expressing cells, S100A13 overexpression potentiated bradykinin-induced and ionophore-stimulated calcium transients compared with mutant cells. Mechanistically, S100A13 may modulate IP₃ receptor–mediated [Ca²⁺] release from the ER [25,26]. S100A13 may also regulate [Ca²⁺] influx across the plasma membrane, likely through modulation of [Ca²⁺]-permeable channels such as members of the transient receptor potential (TRP) channel family [26,27]. This process may be further reinforced by ER–mitochondrial crosstalk through mitochondria-associated membranes (MAMs), which facilitate efficient mitochondrial [Ca²⁺] uptake and dynamic calcium exchange between the ER and mitochondria [25,26].

Intriguingly, in this study, we observed upregulation of MFN1/2 and OPA1 expression, accompanied by reduced MFF expression, in both wild-type and p.I80Gfs*13 mutant S100A13–expressing cells, despite markedly different mitochondrial phenotypes. Wild-type S100A13 promoted a fusion-competent mitochondrial network, whereas the p.I80Gfs*13 mutant induced mitochondrial fragmentation and a shift toward a fission-dominant phenotype. The shared amino acid sequence (residues 1–79) retained in both wild-type and p.I80Gfs*13 mutant S100A13 may partially contribute to the observed regulation of mitochondrial dynamics–related protein expression.

A central finding of this study is the association between S100A13-mediated calcium regulation and OPA1 functional activity in both wild-type and p.I80Gfs*13 mutant S100A13–expressing cells. OPA1 exists as two functional isoforms: the long isoform (L-OPA1), which mediates IMM fusion and maintains cristae integrity, and the short isoform (S-OPA1), which is generated through proteolytic processing and is commonly associated with mitochondrial stress responses [28,29]. OPA1 activity is tightly dependent on mitochondrial bioenergetic status and maintenance of ΔΨm [30]. Loss of ΔΨm is known to activate the stress-responsive mitochondrial metalloprotease OMA1, which promotes proteolytic cleavage of l-OPA1 into S-OPA1 under conditions of mitochondrial dysfunction [16]. Excessive OPA1 processing disrupts cristae organization and impairs IMM fusion [16,30]. Although OPA1 isoform processing was not directly assessed in the present study, the observed reduction in ΔΨm and disruption of cristae integrity in mutant cells are consistent with stress-associated OMA1-mediated impairment of l-OPA1 functional activity. The S100A13 p.I80Gfs*13 mutant disrupted intracellular [Ca²⁺] homeostasis and reduced ΔΨm, thereby creating a bioenergetically unfavorable environment that limits functional l-OPA1 activity. In contrast, preserved intracellular calcium transients in wild-type S100A13–expressing cells were associated with maintenance of ΔΨm. Preservation of ΔΨm in wild-type cells may support physiological YME1L-mediated OPA1 processing. In addition to OPA1 upregulation, S100A13 p.I80Gfs*13–expressing cells exhibited increased expression of MFN1/2. MFN1 and MFN2 play essential roles in regulating mitochondrial outer membrane fusion. MFN2 is critically involved in ER–mitochondrial tethering and calcium exchange, processes essential for maintenance of mitochondrial bioenergetic homeostasis [[6], [7], [8]]. Impairment of intracellular calcium homeostasis in mutant cells may impair MFN2-associated ER–mitochondrial communication and OMM fusion competency.

Additionally, S100A13 p.I80Gfs*13–expressing cells exhibited reduced MFF expression. MFF, a mitochondrial fission factor, plays an important role in recruitment of DRP1 to the mitochondrial outer membrane, thereby facilitating mitochondrial fission [12]. Mitochondrial fission is tightly regulated by calcium-dependent DRP1 activation pathways, including calcineurin-mediated signaling [11,13]. However, DRP1 activation and mitochondrial recruitment were not directly evaluated in the present study. Impairment of intracellular calcium homeostasis in mutant cells may further disrupt MFF-associated mitochondrial fission signaling, resulting in excessive mitochondrial fragmentation and heterogeneous mitochondrial morphology. Based on these findings, our data suggest that S100A13-mediated intracellular [Ca²⁺] homeostasis is an important regulator of mitochondrial dynamics. Mitochondrial dynamics are not regulated solely by mitochondrial fusion–fission proteins. Rather, multiple regulatory layers are required for proper regulation of mitochondrial dynamics, consistent with previous studies [31]. Previous studies have demonstrated that mitochondrial dynamics are tightly regulated by metabolic and cellular stress status [19,31,32].

Many diseases are associated with disruption of mitochondrial structure, which is closely linked to cellular stress responses, inflammation, and release of mitochondrial damage-associated molecular patterns (mtDAMPs) [33,34]. In the present study, we demonstrated that wild-type S100A13 promotes mitochondrial fusion and supports maintenance of mitochondrial structural integrity. These findings suggest a protective role of wild-type S100A13 in preserving MQC. Importantly, these findings have direct relevance to pulmonary disease, where mitochondrial dysfunction contributes to epithelial injury and disease progression [22,23]. Our study provides a mechanistic framework linking S100A13 to calcium-dependent regulation of mitochondrial dynamics in lung epithelial cells. The present findings provide a mechanistic basis for therapeutic strategies targeting mitochondrial dysfunction in pulmonary diseases. Moreover, this concept is supported by recent studies identifying calcium signaling and mitochondrial integrity as emerging therapeutic targets in chronic disorders, including heart failure, ischemic heart disease, and arrhythmias [23,24,35,36].

To our knowledge, this study is the first to identify S100A13, particularly its C-terminal EF-hand domain, as a calcium-dependent upstream regulator linking intracellular [Ca²⁺] homeostasis to mitochondrial dynamics in lung epithelial cells. Despite these advances, several limitations should be acknowledged. First, this study was conducted in an in vitro epithelial cell model, which may not fully recapitulate the complexity of in vivo systems. Second, although our findings support a calcium-dependent regulatory mechanism, the precise molecular intermediates linking S100A13 to mitochondrial regulatory pathways remain unclear. In particular, the potential involvement of [Ca²⁺]-dependent enzymes such as calcineurin and their role in DRP1 activation warrants further investigation. Future studies should further elucidate OPA1 isoform processing and OMA1/YME1L regulatory balance underlying S100A13-mediated regulation of mitochondrial dynamics. Finally, in vivo validation and studies in patient-derived samples are necessary to establish the translational relevance of S100A13-mediated mitochondrial regulation in pulmonary disease.

## 5 Conclusion

In summary, we identified S100A13 as a critical regulator of mitochondrial architecture in lung epithelial cells through its capacity to modulate intracellular [Ca²⁺] mobilization. Our findings demonstrate that wild-type S100A13 sustains [Ca²⁺]-dependent signaling required for maintenance of ΔΨm, thereby preserving functional L-OPA1 activity and cristae structural integrity. In addition, wild-type S100A13 was associated with increased expression of MFN1/2 and OPA1, accompanied by reduced MFF expression, supporting regulation of mitochondrial fusion–fission dynamics. In contrast, the p.I80Gfs*13 mutant disrupted intracellular [Ca²⁺] homeostasis, reduced ΔΨm, impaired mitochondrial structural integrity, and promoted mitochondrial fragmentation.

## Funding

This work was supported by 10.13039/501100002383King Saud University and 10.13039/501100002382King Faisal Specialist Hospital and Research Centre (KFSH&RC), Riyadh, Saudi Arabia (Study No. 2250005).

## CRediT authorship contribution statement

**Somaya Alqattan:** Conceptualization, Methodology, Validation, Investigation, Data curation, Formal analysis, Visualization, Writing – original draft. **Mona Alonazi:** Supervision, Methodology, Investigation, Validation, Writing – review & editing. **Eid Almutairy:** Conceptualization, Supervision, Validation, Writing – review & editing. **Vineesh V. Raveendran:** Methodology, Data curation, Writing – review & editing. **Abdulaziz Alamri:** Conceptualization, Formal analysis, Validation, Data curation. **Futwan Al-mohanna:** Conceptualization, Supervision, Data curation, Writing – review & editing.

## Declaration of competing interest

Eid Al-Mutairy and Futwan Al-Muhanna are listed as inventors on a patent entitled *“Method for Diagnosing or Treating Pulmonary Fibrosis Using S100A13 Protein.*” The remaining authors declare no competing interests.

## Data Availability

Data will be made available on request.

## Appendix Supplementary materials

Image, application 1

Image, application 2
