# Supplementary material for: Role of recombinant S100A13 expression in regulating mitochondrial fission and fusion in lung epithelial cells
Source: BBA Adv. 2026 Jun 4;10:100195. doi: 10.1016/j.bbadva.2026.100195 (PMC13276454; doi:10.1016/j.bbadva.2026.100195)
Supplement: Supplementary file 1 [file mmc1.docx]

**
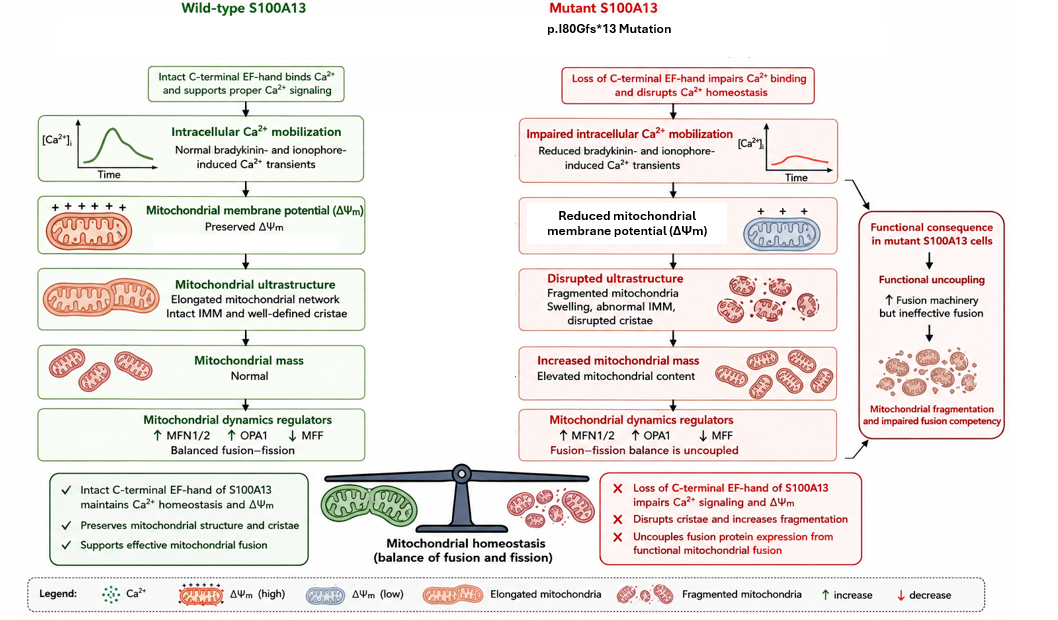
**

**Supplementary Figure: Proposed Mechanistic Model of S100A13-Mediated Regulation of Mitochondrial Dynamics in BEAS-2B Cells**

Schematic illustration summarizing the differential effects of wild-type and mutant S100A13 on intracellular [Ca²⁺] homeostasis, mitochondrial membrane potential (ΔΨm), mitochondrial ultrastructure, and mitochondrial dynamics in BEAS-2B cells. In wild-type S100A13–expressing cells, the intact C-terminal EF-hand domain maintains intracellular [Ca²⁺] mobilization and preserves ΔΨm, thereby supporting mitochondrial ultrastructural integrity, preserved cristae architecture, balanced mitochondrial fusion–fission dynamics, and effective mitochondrial fusion competency. In contrast, the S100A13 p.I80Gfs*13 mutation disrupts the C-terminal EF-hand domain, leading to impaired intracellular [Ca²⁺] mobilization, mitochondrial depolarization, abnormal mitochondrial ultrastructure, cristae disruption, increased mitochondrial mass, and mitochondrial fragmentation. Although mutant S100A13 increased expression of fusion-associated proteins (MFN1/2 and OPA1) and reduced expression of the mitochondrial fission mediator MFF, these molecular alterations were not accompanied by effective mitochondrial fusion, indicating functional uncoupling between fusion protein expression and mitochondrial fusion competency. Overall, these findings support a critical role for the C-terminal EF-hand domain of S100A13 in maintaining mitochondrial structural integrity and mitochondrial dynamics through regulation of intracellular [Ca²⁺] homeostasis.
