# Supplementary material for: Role of recombinant S100A13 expression in regulating mitochondrial fission and fusion in lung epithelial cells
Source: BBA Adv. 2026 Jun 4;10:100195. doi: 10.1016/j.bbadva.2026.100195 (PMC13276454; doi:10.1016/j.bbadva.2026.100195)
Supplement: Supplementary file 2 [file mmc2.docx]

**Appendix A. Supplementary Data**

**
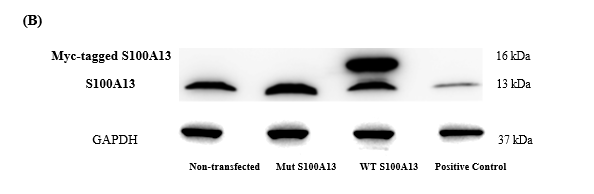

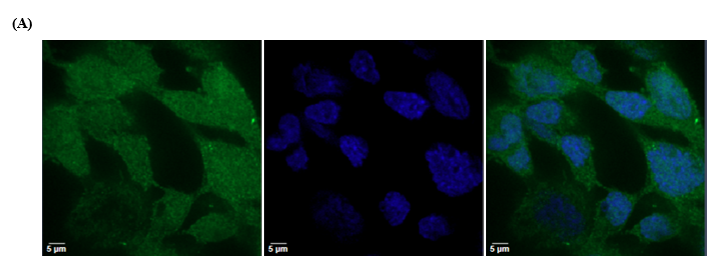
**

**
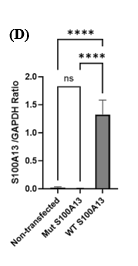

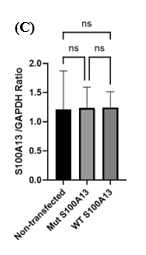
**

**Supplementary Figure S1: Expression of S100A13 in BEAS-2B Lung Epithelial Cells**

(A) Immunofluorescence analysis of endogenous S100A13 expression in BEAS-2B cells. S100A13 is shown in green, nuclei are counterstained with DAPI (blue). Scale bar: 5 µm. (B) Western blot analysis of endogenous and exogenous S100A13 expression. Non-transfected and mutant S100A13–expressing cells (Mut S100A13) display a single band corresponding to endogenous S100A13 (~13 kDa). In contrast, wild-type S100A13–expressing cells (WT S100A13) exhibit an additional band at ~16 kDa, consistent with Myc-tagged S100A13. HeLa cell lysate was used as a positive control. GAPDH (~37 kDa) was used as a loading control. (C-D) Densitometric quantification of endogenous (C) and exogenous (D) S100A13 expression normalized to GAPDH. Endogenous S100A13 levels were not significantly different among groups (p > 0.05), whereas exogenous S100A13 expression was significantly increased in WT S100A13–expressing cells compared with Mut S100A13 and non-transfected controls (**** p ≤ 0.0001).

Data are presented as mean ± SD from at least three independent experiments. Statistical analysis was performed using one-way ANOVA followed by Tukey’s multiple comparisons test.

**
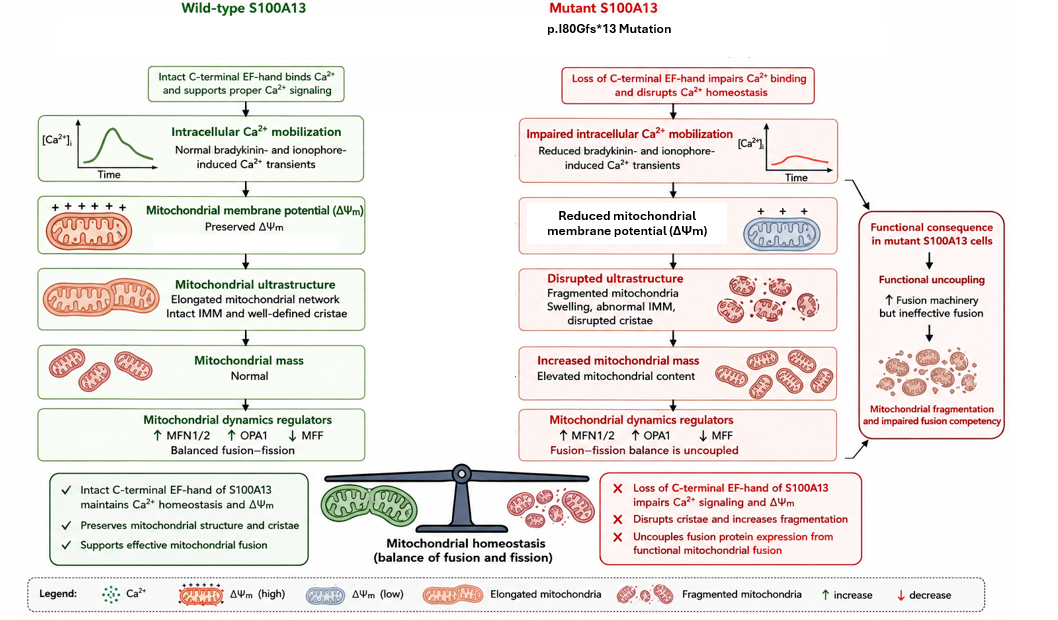
**

**Supplementary Figure S2: Proposed Mechanistic Model of S100A13-Mediated Regulation of Mitochondrial Dynamics in BEAS-2B Cells**

Schematic illustration summarizing the differential effects of wild-type and mutant S100A13 on intracellular [Ca²⁺] homeostasis, mitochondrial membrane potential (ΔΨm), mitochondrial ultrastructure, and mitochondrial dynamics in BEAS-2B cells. In wild-type S100A13–expressing cells, the intact C-terminal EF-hand domain maintains intracellular [Ca²⁺] mobilization and preserves ΔΨm, thereby supporting mitochondrial ultrastructural integrity, preserved cristae architecture, balanced mitochondrial fusion–fission dynamics, and effective mitochondrial fusion competency. In contrast, the S100A13 p.I80Gfs*13 mutation disrupts the C-terminal EF-hand domain, leading to impaired intracellular [Ca²⁺] mobilization, mitochondrial depolarization, abnormal mitochondrial ultrastructure, cristae disruption, increased mitochondrial mass, and mitochondrial fragmentation. Although mutant S100A13 increased expression of fusion-associated proteins (MFN1/2 and OPA1) and reduced expression of the mitochondrial fission mediator MFF, these molecular alterations were not accompanied by effective mitochondrial fusion, indicating functional uncoupling between fusion protein expression and mitochondrial fusion competency. Overall, these findings support a critical role for the C-terminal EF-hand domain of S100A13 in maintaining mitochondrial structural integrity and mitochondrial dynamics through regulation of intracellular [Ca²⁺] homeostasis.
